# Histological and Histochemical Changes Induced by Alkylating Agents in Transplanted Rat Sarcoma with Special Reference to Sarcolysine

**DOI:** 10.1038/bjc.1960.7

**Published:** 1960-03

**Authors:** M. A. Presnov

## Abstract

**Images:**


### HISTOLOGICAL AND HISTOCHEMICAL CHANGES INDUCED BY ALKYLATING AGENTS IN TRANSPLANTED RAT SARCOMA WITH SPECIAL REFERENCE TO SARCOLYSINE

M. A. PRESNOV

*From the Department of Experimental Chemotherapy of the Institute of Experimental and Clinical Oncology. Academy of Medical Sciences of the U.S.S.R., Moscow.*

Received for publication January 20, 1960

SINCE substances with antineoplastic activity became available, particularly those known as biological alkylating agents, systematic histological and cytological studies of their action have been made (Yoshida, 1952 ; Jdanov, 1955 ; Koller and Veronesi, 1956 ; Kellner, 1957 ; Breuvis, 1958 ; Koller, 1958 ; Larionov and Presnov, 1958 ; Presnov and Spasskaya, 1959).

In this report the histological and histochemical changes that occur in several experimental tumours following the administration of particular alkylating agents are described.

### MATERIALS AND METHODS

Three transplanted rat tumours were used.

*Sarcoma* 45.—This is a rat tumour of Russian origin. It is a malignant spindle-cell tumour, transplantation of which is successful in 100 per cent of animals, and which kills its host in 25 to 30 days. This tumour regresses completely with sarcolysine, dopan and ethylene-imino-benzoquinone (E39).

*Sarcoma M*-1.—This is a malignant, polymorphous-cell tumour of Russian origin. On transplantation 100 per cent of grafts are successful, and the tumour kills its host in 30 days. Sarcoma M-1 is more resistant to chemotherapy than Sarcoma 45.

*Yoshida sarcoma.*—This well-known tumour was first introduced into the Soviet Union in 1957.

All three tumours were transplanted subcutaneously into albino rats of either sex weighing between 80 and 100 g. The Yoshida sarcoma was also studied in the ascites form. 633 rats were used.

Chemotherapy was begun when the transplanted tumours were 7 to 10 days old, measured 0·7 to 1·0 cm. in diameter, and weighed 0·5 to 1·0 g. In the case of the ascites tumour, treatment was begun 4 days after transplantation.

Three alkylating agents were used : sarcolysine, *p*-di-(2-chloroethyl)amino-DL-phenylalanine, (Larionov, Khokhlov, Schkodinskaya, Vasina, Truscheikina, Novikova-Smirnova, 1955 ; Truscheikina, 1956 ; Larionov, 1959) known as merphalan in Great Britain (Bergel and Stock, 1953, 1954 ; Bergel, Burnop and Stock, 1955) ; dopan, 5-di-(2-chloroethyl)amino-4-methyl-uracil (Larionov and Platonova, 1955 ; Platonova, 1957 ; Larionov, 1959) ; ethylene-imino-benzoquinone, E.39, 2, 5-diethylenimino-3,6-dipropoxy-1,4-benzoquinone (Domagk, Peterson, and Gauss, 1954 ; Domagk, 1958).

Rats bearing Sarcoma 45 received either sarcolysine or dopan ; those bearing Sarcoma M-1 received only sarcolysine, while rats bearing the Yoshida tumour received each of the three compounds. The doses and routes of administration of the drugs are shown in Table I.

TABLE I.—*Doses and Routes of Administration of Alkylating Agents in Transplated Rat Tumours.*

| Tumour | Sarcolysine | Dopan | E39 |
|---|---|---|---|
| Sarcoma M-1 | Intraperitoneal<br>5 mg. per kg. 5 to 7 times, 3-day intervals<br>10 mg. per kg. 5 to 7 times at 3-* or 7-day intervals | — | — |
| Sarcoma 45 | 15 mg. per kg. once or in 3–5 divided doses 5 mg. per kg. at 3-day intervals | Oral<br>0·3 mg. per kg. daily for 15 days, or 0·75 mg. per kg. 7–9 times every 3 days | — |
| Yoshida solid | Intraperitoneal<br>1·5 mg. per kg. daily for 20 days | Oral<br>0·3 mg. per kg. daily for 20 days | Intraperitoneal<br>0·6 mg. per kg. daily for 20 days. |
| Yoshida ascites | As above, or orally | — | — |

* Some rats died.

The histological and histochemical methods used were as follows :

(a) Paraffin-embedded material fixed in 10 per cent neutral formol saline. Haematoxylin and eosin, Feulgen's method for deoxyribonucleic acid, methyl-green-pyronin for ribonucleic acid, Van Gieson's stain for collagen, Foot's stain for reticulin, and toluidine blue for metachromasia.

(b) Frozen sections of material fixed in 10 per cent neutral formol saline. Sudan III for neutral fat.

(c) Paraffin-embedded material fixed in cold 80 per cent ethanol. Gomori's method for alkaline phosphatase. The incubation time was between 2 and 5 hours (Presnov, 1954, 1956).

(d) Paraffin-embedded material fixed by Schabadasch's method (1949). McManus's modification of Hotchkiss's periodic-acid-Schiff (PAS) method for polysaccharide.

(e) Methanol-fixed smears of ascitic fluid. May-Grünwald-Giemsa.

(f) Vital fluorescence microscopy was carried out in collaboration with Dr. P. V. Breuvis to study the nucleoproteins.

To allow for artefacts in staining, the treated and untreated animals were killed at the same time, and the tissues from both were handled side by side throughout each procedure, so that every preparation from a treated tumour could be compared directly with its counterpart from an untreated tumour which had been subjected to identical manipulation.

### RESULTS

Sarcoma 45 regressed in every animal under treatment with sarcolysine and dopan, but Sarcoma M-1 regressed completely only after administration of sarcolysine at high dosage (five to seven injections at 3-day intervals of 10 mg. per kg. of body weight). Subcutaneous nodules of the Yoshida sarcoma regressed completely under therapy with sarcolysine or dopan in only 25 per cent of the rats, and in only 12 per cent when E39 was given. Sarcolysine always caused complete regression in the ascitic form of Yoshida sarcoma, whether oral or intraperitoneal routes were employed.

The histological and histochemical changes during the course of regression were similar in all three tumours and with each of the three drugs.

*General histological changes.*—During the course of regression, striking changes were observed both in the parenchyma and stroma of the tumours. The earliest changes, decrease in mitotic activity and nuclear fragmentation, were observed within 1 hour of the intraperitoneal injection of sarcolysine in the case of the Yoshida ascites sarcoma, and in 3 hours in the case of the solid sarcomata, or in the ascites sarcoma after oral administration. Mitotic activity disappeared if treatment was continued. Cells in prophase were the first to disappear. Cell destruction accompanied the decrease in mitotic activity, their nuclei became angular and the chromatin coarsened. Pyknotic changes were less common. Abnormal multipolar mitoses appeared. This sequence of changes is illustrated in Fig. 1–6, 16–24. A very characteristic change was the enlargment of many of the cells, which has been frequently described as a feature of the action of alkylating agents. With further decrease in mitotic activity, bizarre multinucleated giant cells appeared, presumably as a result of amitotic division (Fig. 2, 6, 17, 18). During treatment the nucleoli of the tumour cells increased in size, showed increased pyroninophilia and alkaline phosphatase activity.

The stromal and parenchymal changes described did not occur simultaneously throughout the tumour but were patchy and irregular. In the Yoshida ascites cells the changes were comparable with those seen in the solid tumours, and included the appearance of pathological mitoses, the formation of multinucleated cells and marked enlargement of the cells and their nuclei and nucleoli (Fig. 22–24).

*Changes in the nucleoproteins.*—The first change observed was droplet formation within the nuclei (Fig. 5). The droplets stained deeply with Feulgen's stain and also showed alkaline phosphatase activity. The nuclei of the greatly enlarged cells were Feulgen negative almost always, but rarely gave a positive reaction.

Studies on the nucleoproteins in this material, by means of vital fluoresence microscopy showed that changes occurred 3 to 6 hours after the first treatment with sarcolysine, and consisted of increased fluoresence in the nuclei of affected cells (Breuvis, 1958).

*Fatty change.*—Fat is seen only in small amounts in necrotic areas of the untreated tumours, but appeared in the form of small droplets in the cytoplasm of a majority of the cells 24 to 48 hours after the beginning of treatment. Later all the cells contained fat, and the small droplets fused into larger ones, becoming liberated in the stroma as cells disintegrated. Eventually large quantities of fat accumulated, and this could still be recognised embedded in the scar which remained after the tumour had regressed completely and had become replaced by fibrous tissue.

*Alkaline phosphatase activity.*—In untreated tumours, the enzyme is located mainly in the nuclei and nucleoli of the cells. Following treatment, it appeared also in the cytoplasm, while in fragmented nuclei the amount increased greatly.

Later alkaline phosphatase activity appeared also in the stroma and between the newly formed collagen fibres (Fig. 7, 8. 9).

*Ribonucleic acid.*—Pyroninophilia, sometimes after an initial increase, gradually decreased in the tumour cells, and later disappeared entirely.

*Metachromasia.*—Cytoplasmic metachromasia with toluidine blue also disappeared during treatment.

*Glycogen.*—Glycogen is not present in untreated tumours, except in lymphocytes in the stroma, or in necrotic areas. Glycogen did not appear in the tumour cells as a result of treatment, though pink droplets, not disappearing after treatment with amylase, appeared (McManus-Hotchkiss reaction). They were presumably mucopolysaccharide in nature.

*Stromal reaction.*—The first change was oedema, which separated the tumour cells slightly from one another. Untreated tumours contain very little collagen, but argyrophilic fibres are numerous. During treatment, collagen fibres appeared, became increasingly numerous and eventually formed, a dense scar. During the same period, the argyrophilic fibres lost their affinity for silver stains, became thicker and were presumably transformed into collagen fibres (Fig. 10–15).

### DISCUSSION

Alkylating agents kill cells. Death may be rapid, and is shown by fragmentation of the nuclei, or slow, when death is preceded by the appearance of fat in the cytoplasm, increase in alkaline phosphatase activity, disappearance of ribonnucleic acid and of chromotropic substances from the cytoplasm, and by enlargement of the nuclei and nucleoli. Amitotic division may be a form of dystrophic change in which the cells, though damaged, can survive. Since large doses of alkylating agents can cause complete regression of tumours, it is likely that all the cells are damaged to some extent, and the type and degree of damage may depend on the functional activity of the cell at the time of exposure, some states of activity rendering the cell more vulnerable than others. The drugs studied appear to affect the process of cell division and also to interfere with growth, the latter effect being relatively small. Sarcolysine appears to suppress the growth of cells more than dopan or E39.

The alkylating agents appear to act on the tumour cells immediately as shown by the rapidity with which visible changes in the cells occur, for instance in the ascites tumours. That nucleoproteins are damaged directly is suggested by the early occurrence of nuclear fragmentation, increased fluorescence and decrease of mitotic activity. It is possible that the exact mechanisms vary from one alkylating agent to another. Powerful agents like sarcolysine might have additional actions.

It is important to emphasize that the stromal reaction follows the changes in the tumour cells, and is perhaps directed towards removal of the damaged tumour cells. It is assumed that increased alkaline phosphatase activity helps in removing fragmented cell components rich in phosphoesters and nucleic acids.

### SUMMARY

1. The histological and histochemical changes accompanying drug-induced regression were followed in three transplanted rat tumours.

2. The tumours were the Sarcoma 45, the Sarcoma M-1, and the Yoshida sarcoma in solid and ascites form.

3. The drugs used were the alkylating agents sarcolysine (merphalan), dopan, and ethylene-imino-benzoquinone (E39).

4. Eleven histological and histochemical methods were used.

5. Striking parenchymal and stromal changes were observed. Early changes included decrease in mitotic activity and nuclear fragmentation, while later, enlargement of surviving cells, and the appearance of multinucleated forms was seen. Fat appeared in the cytoplasm, and ribonucleoprotein and chromotropic substances disappeared. In the stroma, the argyrophilic fibres became converted into collagen. Alkaline phosphatase activity increases first in the cells, and later in the stroma.

6. The alkylating agents appear to act immediately on the tumour cells. The stromal reaction is secondary to the death of the tumour cells. The drugs affect the nucleoproteins directly.

I wish to thank Professor L. F. Larionov for helpful advice.

### REFERENCES

BERGEL, F. AND STOCK, J. A.—(1953) *Rep. Brit. Emp. Cancer Campgn.*, **31**, 6.—(1954) *J. chem. Soc.*, 2409.

---

### EXPLANATION OF PLATES

FIG. 1.—Sarcoma 45. Untreated tumour. Haematoxylin and eosin. ×280.

FIG. 2.—Sarcoma 45. 24 hours after the 3rd intraperitoneal injection of sarcolysine, 5 mg. per kg. of body weight at 72-hour intervals. Haematoxylin and eosin. ×280.

FIG. 3.—Sarcoma 45. 72 hours after the 3rd injection. Haematoxylin and eosin. ×280.

FIG. 4.—Sarcoma 45. Untreated. Feulgen reaction. ×280.

FIG. 5.—Sarcoma 45. 48 hours after sarcolysine, 5 mg. per kg. of body weight. Feulgen reaction. ×280.

FIG. 6.—Sarcoma 45. 24 hours after the 3rd intraperitoneal injection of sarcolysine, 5 mg. per kg. of body weight at 72-hour intervals. Feulgen reaction. ×280.

FIG. 7.—Sarcoma 45. Untreated. Gomori reaction for alkaline phosphatase. ×280.

FIG. 8. Sarcoma 45. 24 hours after the 2nd intraperitoneal injection of sarcolysine. Gomori reaction. ×280.

FIG. 9.—Sarcoma 45. 72 hours after the 3rd injection. Gomori reaction. ×280.

FIG. 10.—Sarcoma 45. Untreated. Van Gieson. ×280.

FIG. 11.—Sarcoma 45. 72 hours after the 2nd intraperitoneal injection of sarcolysine. Van Gieson. ×280.

FIG. 12.—Sarcoma 45. 72 hours after the 3rd intraperitoneal injection. Van Gieson. ×280.

FIG. 13.—Sarcoma 45. Untreated. Foot. ×280.

FIG. 14.—Sarcoma 45. 24 hours after the 3rd intraperitoneal injection of sarcolysine. Foot. ×280.

FIG. 15.—Sarcoma 45. 72 hours after the 3rd intraperitoneal injection. Foot. ×280.

FIG. 16.—Sarcoma M-1. Untreated. Haematoxylin and eosin. ×280.

FIG. 17.—Sarcoma M-1. 48 hours after 2 injections of sarcolysine, 10 mg. per kg. of body weight at 73-hour intervals. Haematoxylin and eosin. ×280.

FIG. 18.—Sarcoma M-1. 48 hours after 5th injection of sarcolysine, 10 mg. per kg. of body weight at 72-hour intervals. Haematoxylin and eosin. ×280.

FIG. 19.—Sarcoma M-1. Untreated. Feulgen. ×280.

FIG. 20.—Sarcoma M-1. 48 hours after the 3rd injection of sarcolysine. Feulgen. ×280.

FIG. 21.—Sarcoma M-1. 48 hours after the 5th injection. Feulgen. ×280.

FIG. 22.—Yoshida ascites tumour. Untreated. May-Grünwald-Giemsa. ×630.

FIG. 23.—Yoshida ascites tumour. 24 hours after the intraperitoneal injection of sarcolysine, 1·5 mg. per kg. of body weight. May-Grünwald-Giemsa. ×630.

FIG. 24.—Yoshida ascites tumour. 24 hours after 3 daily intraperitoneal injections of sarcolysine, 1·5 mg. per kg. of body weight. May-Grünwald-Giemsa. ×630.

1

2

3

4

5

6

Presnov.

7

8

9

10

11

12

13

14

15

16

17

18

Presnov.

19

20

21

22

23

24

Presnov.

*Idem*, BURNOP, V. C. E. AND STOCK, J. A.—(1955) *Ibid.*, 1223.
BREUVIS, P. V.—(1958) *Arkh. Pat.*, **20**, 39.
DOMAGK, G.—(1958) *Ann. N.Y. Acad. Sci.*, **68**, 1197.
*Idem*, PETERSON, S. AND GAUSS, W. A.—(1954) *Z. Krebsforsch.*, **59**, 617.
KELLNER, B.—(1957) *Acta Morph. Acad. Sci., Hung.*, **7**, 4.
KOLLER, P. C.—(1958) *Ann. N.Y. Acad. Sci.*, **68**, 783.
*Idem* AND VERONESI, U.—(1956) *Brit. J. Cancer*, **10**, 703.
JDANOV, G. L.—(1955) *Probl. Oncol.*, **6**, 94.
LARIONOV, L. F.—(1959) *Acta Un. int. Cancr.*, **15**, 42.
*Idem*, KHOKHLOV, A. S., SCHKODINSKAYA, E. N., VASINA, O. S., TRUSCHEIKINA, V. I., NOVIKOVA-SMIRNOVA, M. A.—(1955) *Bull. exp. Biol. Med.*, **1**, 48.
*Idem* AND PLATONOVA, G. N. (1955) *Probl. Oncol.*, **5**, 36.
*Idem* AND PRESNOV, M. A.—(1958) *Arkh. Pat.*, **20**, 32.
PLATONOVA, G. N.—(1957) *Bull. exp. Biol. Med.*, **6**, 53.
PRESNOV, M. A.—(1954) *J. gen. Biol.*, **5**, 321.—(1956) *Probl. Oncol.*, **4**, 423.
*Idem* AND SPASSKAYA, I. G.—(1959) *Ibid.*, **7**, 38.
SCHABADASCH, A. L.—(1949) ' Problems Connected with the Histochemical Investigation of Glycogen in the Normal Nervous System. Study on the Biological Characteristic of and Differences Between Neurones '. Moscow (Medgiz).
TRUSCHEIKINA, V. I.—(1956) *Probl. Oncol.*, **2**, 222.
YOSHIDA, T.—(1952) *J. nat. Cancer Inst.*, **12**, 947.


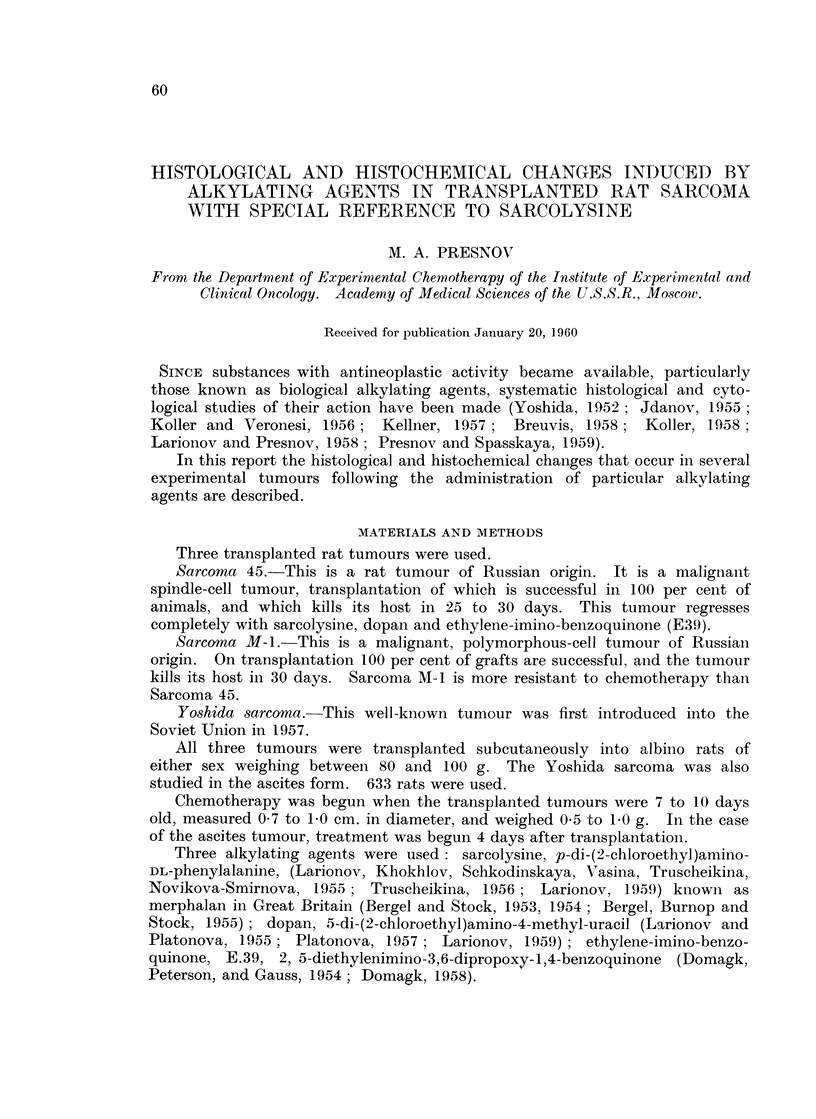

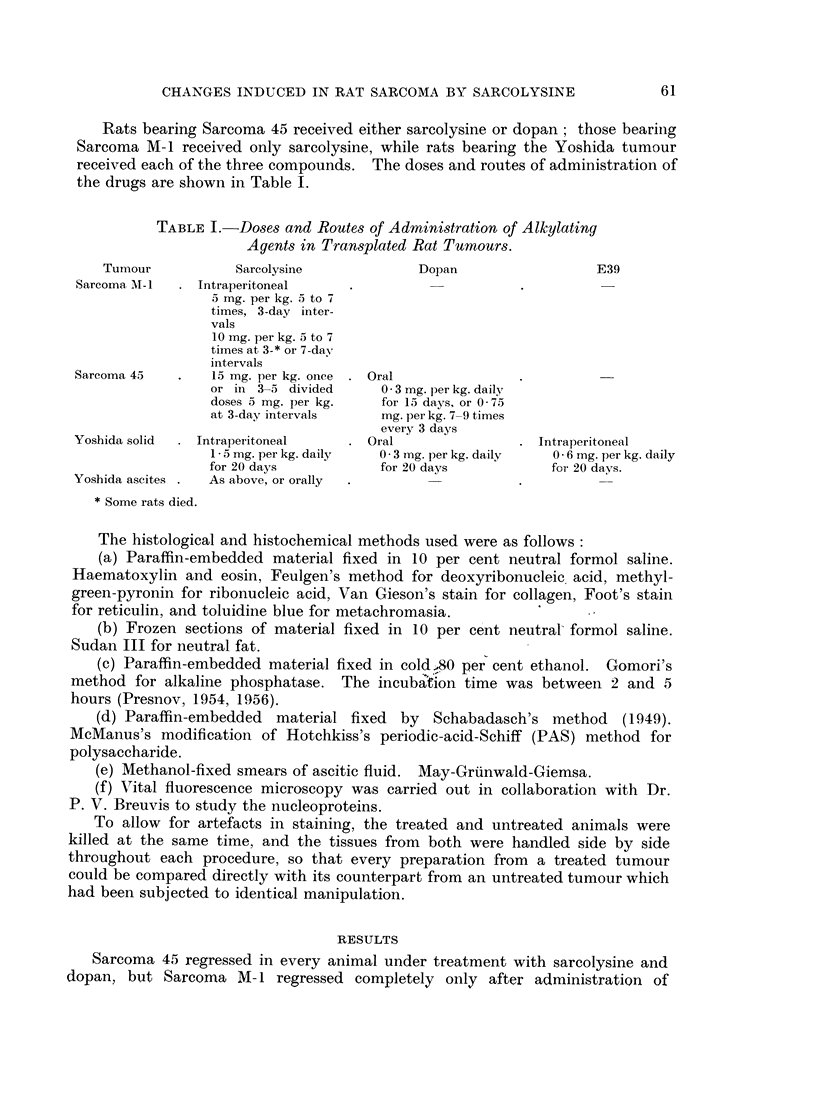

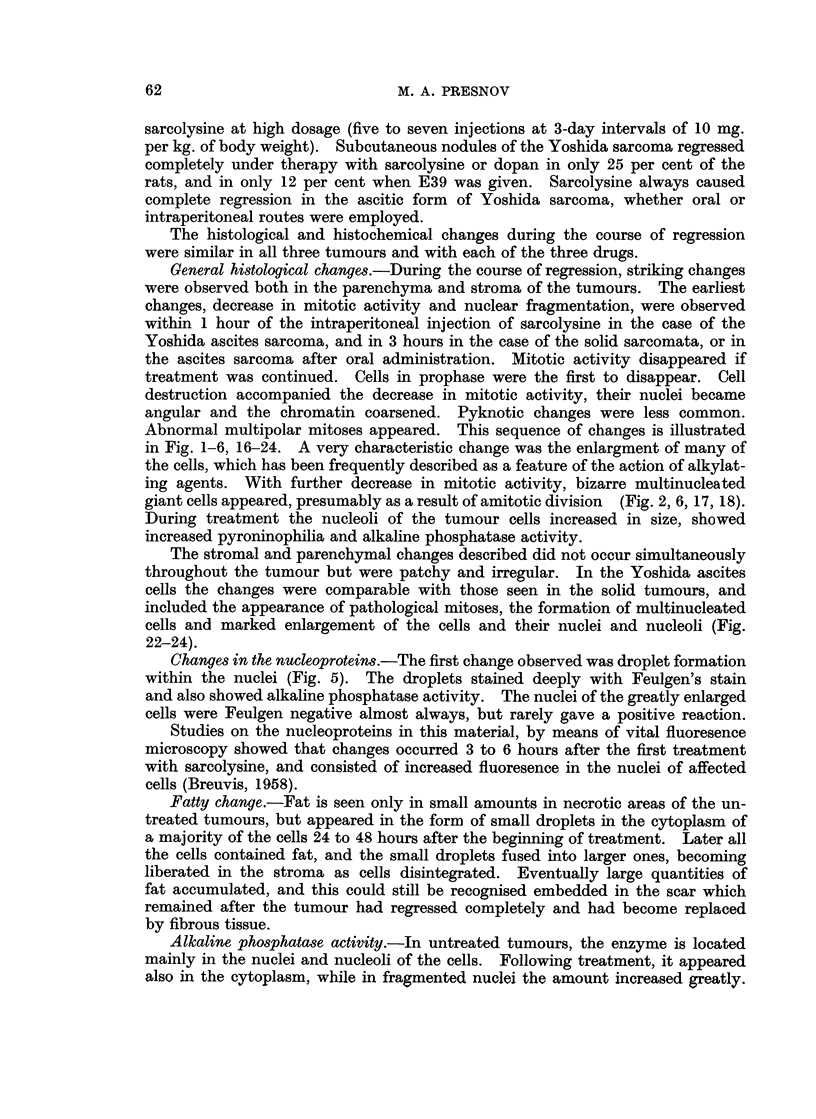

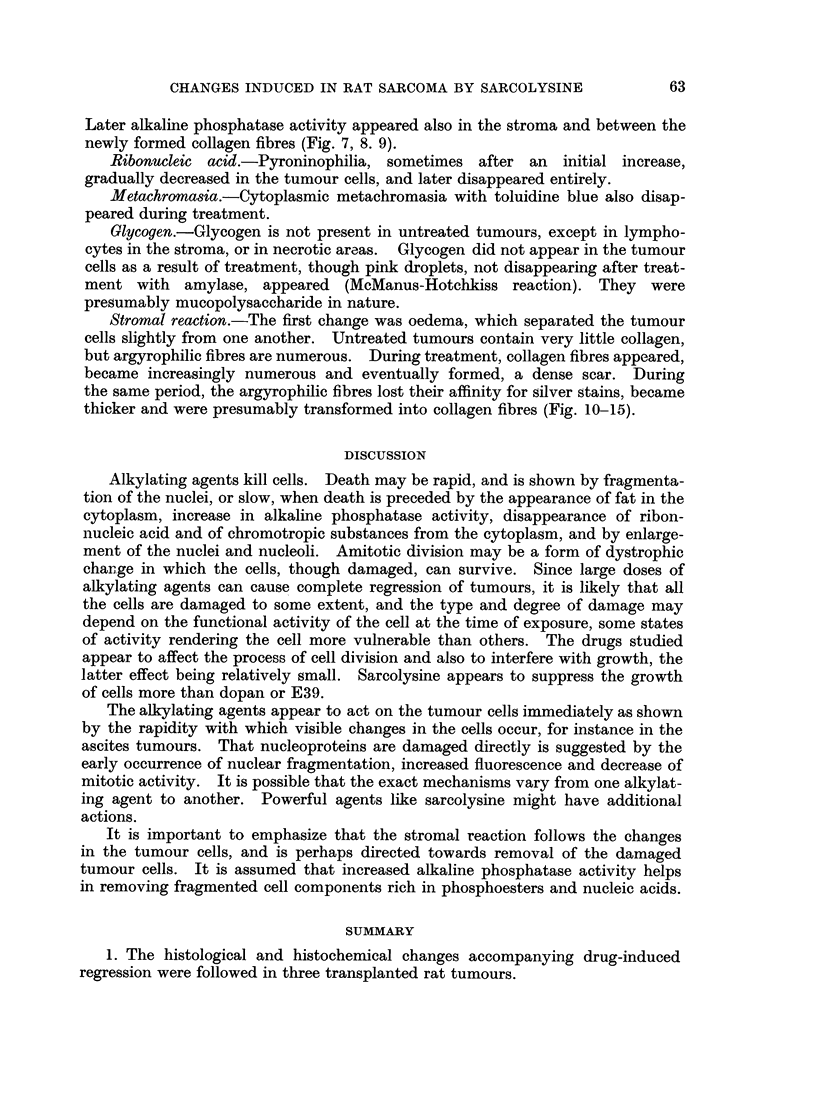

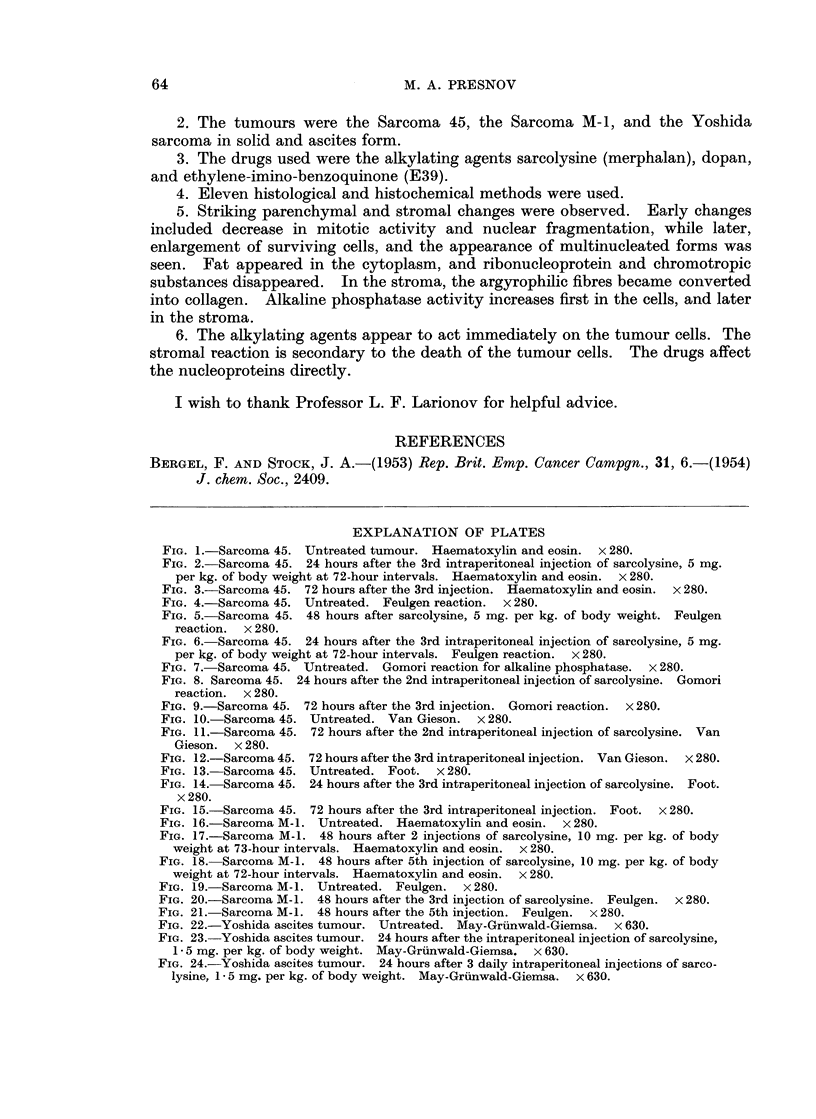

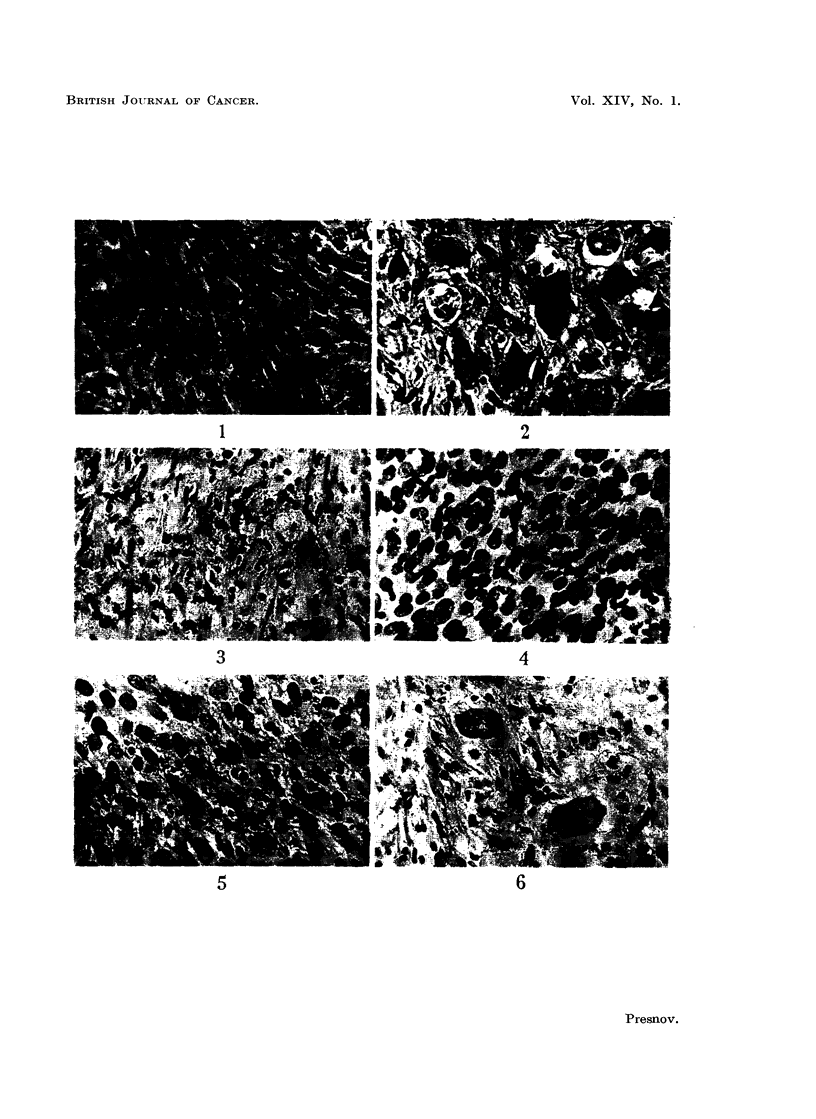

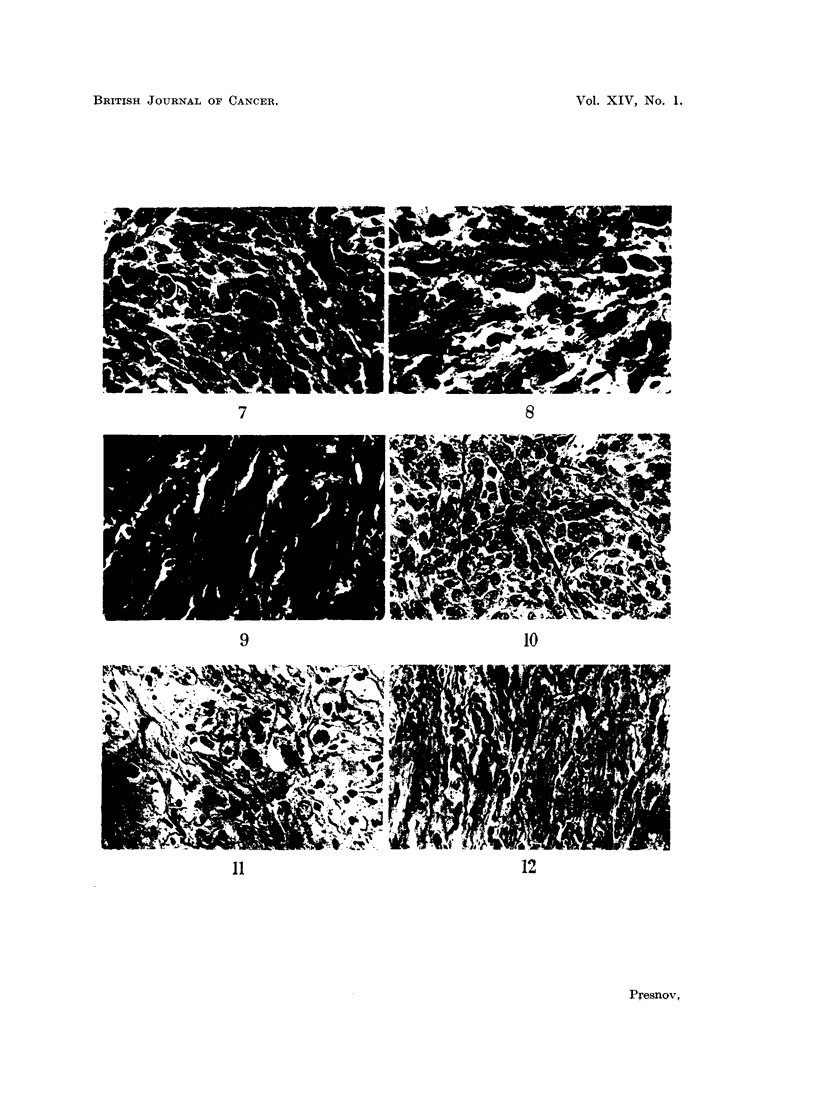

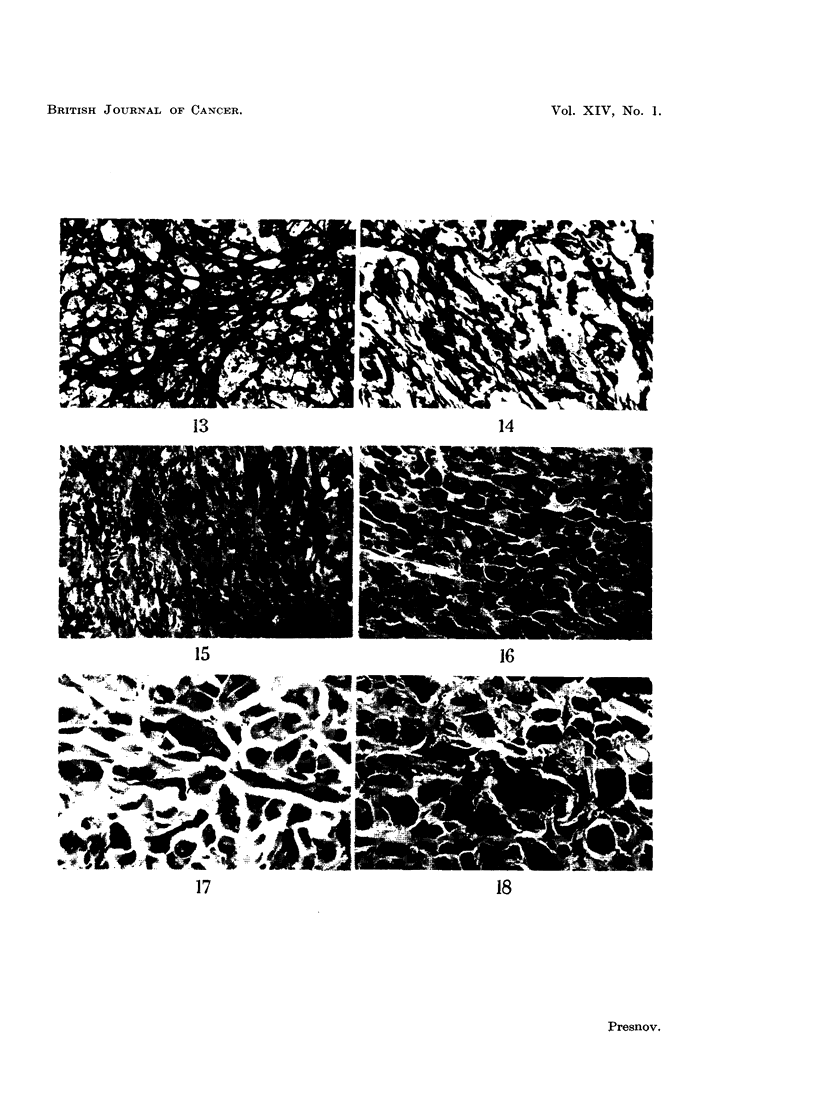

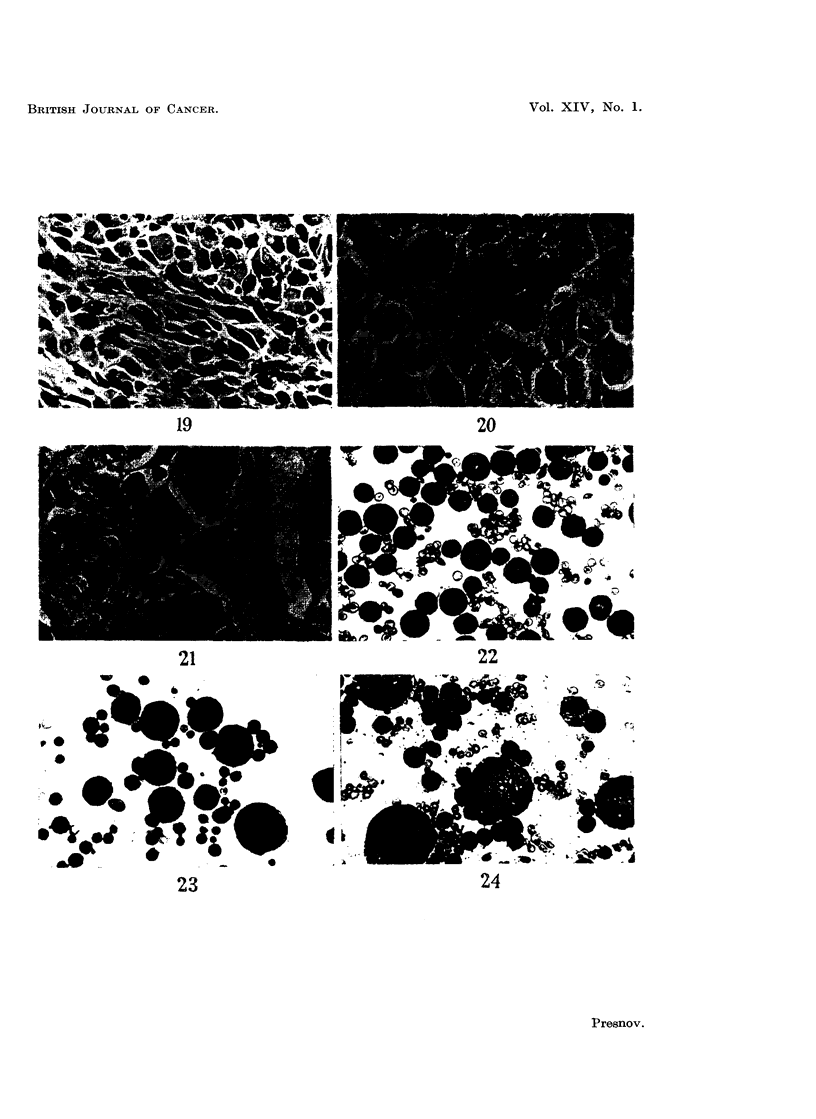

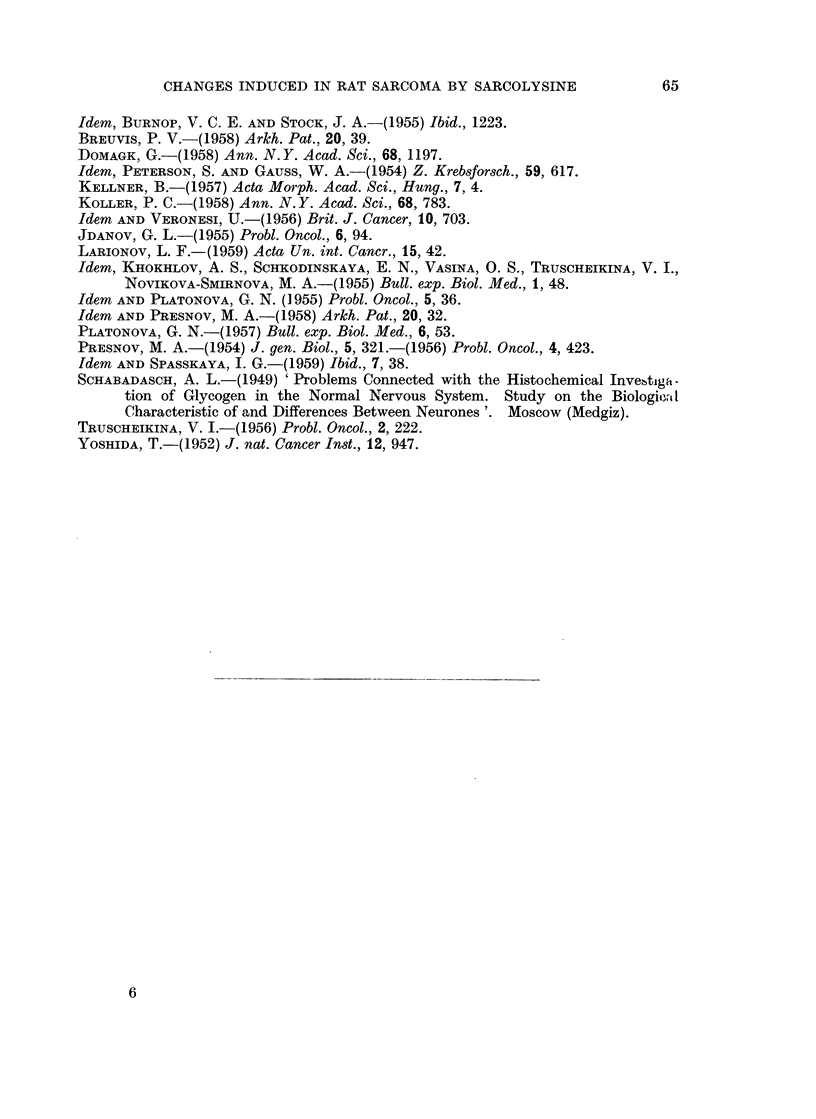


## References

[OCR_00447] DOMAGK G., PETERSEN S., GAUSS W. (1954). Ein Beitrag zur experimentellen Chemotherapie der Geschwüste.. Z Krebsforsch.

[OCR_00450] KOLLER P. C. (1958). Comparative effects of alkylating agents on cellular morphology.. Ann N Y Acad Sci.

[OCR_00471] YOSHIDA T. (1952). Studies on an ascites (reticuloendothelia cell?) sarcoma of the rat.. J Natl Cancer Inst.

